# Predicting excess cost for older inpatients with clinical complexity: A retrospective cohort study examining cognition, comorbidities and complications

**DOI:** 10.1371/journal.pone.0193319

**Published:** 2018-02-23

**Authors:** Kasia Bail, Brian Draper, Helen Berry, Rosemary Karmel, John Goss

**Affiliations:** 1 Health Research Institute, University of Canberra, Canberra, Australia; 2 School of Psychiatry University of New South Wales, Academic Department for Old Age Psychiatry, Prince of Wales Hospital, Sydney, Australia; 3 Professor of Climate Change and Mental Health, Sydney School of Public Health, Sydney Medical School, University of Sydney, Sydney, Australia; 4 Australian Institute of Health and Welfare, Canberra, Australia; Augusta University, UNITED STATES

## Abstract

**Background:**

Hospital-acquired complications increase length of stay and contribute to poorer patient outcomes. Older adults are known to be at risk for four key hospital-acquired complications (pressure injuries, pneumonia, urinary tract infections and delirium). These complications have been identified as sensitive to nursing characteristics such as staffing levels and level of education. The cost of these complications compared to the cost of admission severity, dementia, other comorbidities or age has not been established.

**Method:**

To investigate costs associated with nurse-sensitive hospital-acquired complications in an older patient population 157,178 overnight public hospital episodes for all patients over age 50 from one Australian state, 2006/07 were examined. A retrospective cohort study design with linear regression analysis provided modelling of length-of-stay costs. Explanatory variables included patient age, sex, comorbidities, admission severity, dementia status, surgical status and four complications. Extra costs were based on above-average length-of-stay for each patient’s Diagnosis Related Group from hospital discharge data.

**Results:**

For adults over 50 who have length of stay longer than average for their diagnostic condition, comorbid dementia predicts an extra cost of A$874, (US$1,247); any one of four key complications predicts A$812 (US$1,159); each increase in admission severity score predicts A$295 ($US421); each additional comorbidity predicts A$259 (US$370), and for each year of age above 50 predicts A$20 (US$29) (all estimates significant at *p*<0.0001).

**Discussion:**

Hospital-acquired complications and dementia cost more than other kinds of inpatient complexity, but admission severity is a better predictor of excess cost. Because complications are potentially preventable and dementia care in hospitals can be improved, risk-reduction strategies for common complications, particularly for patients with dementia could be cost effective.

**Conclusions:**

Complications and dementia were found to cost more than other kinds of inpatient complexity.

## Introduction

Older, complicated patients offer the greatest opportunity for reductions of length of stay and, therefore, cost [1]. Nearly one-half of all Australian hospital beds are occupied by patients over age 65 [2], meaning that ‘acute care’ is increasingly ‘aged care’. Hospitals are shifting from caring for acute episodic illness for patients who are otherwise well, to caring for acute episodic illness of patients with higher levels of comorbidities and a range of functional and cognitive support needs [3]. These complex, older patients can be the most medically challenging, time-consuming and costly patients to treat [4]. However, examining quality and cost of care has been obscured by labelling potentially preventable complications as geriatric syndromes [5] and by confusion between concepts of disability, frailty and comorbidity [6].

Consequently further research to understand elements of high-cost inpatient use is needed [7]. The range of complexity that may be associated with cost of older patient characteristics includes age, admission severity, comorbidity (including dementia) and hospital-acquired complications. Establishing costs for these correlates can be based on direct measures of cost, such as collecting information about resources used at the patient level [8]. This is accurate but highly resource intensive, so many analyses instead use estimations of cost related to length of stay. This is because length of stay is the most visible modifiable component of the expense of hospital care, and has been found to be responsive to medical interventions and alterations in nursing characteristics [9, 10]. This background will summarise and interpret what is known about the impact of patient complexity on hospital cost. A review of studies researching patient-level direct cost is provided, including two different approaches to understand costs: estimated cost associated with length of stay; and length of stay irrespective of cost. There are a range of correlates of excess cost and length of stay: older age, greater comorbidity (including dementia), greater admission severity and hospital-acquired complications. These correlates are discussed below in relation to cost and length of stay.

Older patients are associated with higher costs, but ‘older’ can mean aged above 40 years [11], 50 years [12] or 65 years [13]. Once above one of these ‘older’ age criteria, increasing age is only weakly correlated with further increases in length of stay (LOS) or costs [11, 12, 14]. The minor incremental magnitude of costs associated with increase age once in the ‘older age’ bracket result arguably from decision-making around the decreased ‘margin of benefit’ of further treatment and intervention in regards to patient longevity [12].

Length of stay increases with greater comorbidity [12, 13, 15] and between 85.0% and 95.1% of patients over age 55 present with comorbidities [11, 16]. Though comorbidities do not necessarily increase costs [11, 17, 18], most do, even allowing for other factors such as patient, hospital and procedure characteristics. Each comorbidity has been found to have a unique association with the cost of hospitalisation and length of stay, and their combination is additive: the more comorbidities, the greater the resource use [11].

A key comorbidity is dementia, and it is important to consider diagnosis of dementia when examining age, complications, admission severity/general health and comorbidities and the impact on LOS and cost. Dementia is increasingly common [19], frequently implicates high-care needs in hospital [20] and increases costs [21–23]. When controlling for multiple comorbidities, dementia has a greater impact on total costs than do other comorbidities including diabetes, hypertension, and osteoarthritis [21]. Dementia patients who undergo surgery have higher postoperative complications, particularly for complications that are difficult to identify in their early stages, such as pneumonia and urinary tract infection [24]. Dementia patients have almost twice as many complications as non-dementia patients (whether medical or surgical); dementia patients comprise 22% of of the total bed days for episodes that included an acquired complication despite only accounting for 10% of hospital episodes [22].

Admission severity, or how unwell the patient is, is not uniformly examined in the literature, in part due to limited administrative data captured regarding patient acuity and demands of care. Admission severity has been found to increase the likelihood of all types of adverse events, incremental costs and LOS [17, 25]; and therefore is an important determinant of daily resource consumption and LOS [26]. Severity of illness using the Diagnosis Related Groups (DRG) severity of illness scale revealed a significant contribution to cost [13]. When also considered with social issues, disposition issues and clinical stability, severity of illness explains almost 40% of length of stay [27]. In surgical patients, medication, complications, comorbidity, medical specialty, age and LOS explained 56.2% of the variance in demand for care in terms of cost [28]. The American Society of Anesthesiologists’ (ASA) physical status classification system (ASA Classifications: 1. Healthy person, 2. Mild systemic disease, 3. Severe systemic disease, 4. Severe systemic disease that is a constant threat to life, 5. A moribund person who is not expected to survive without the operation, 6. A declared brain-dead person whose organs) are being removed for donor purposes) has also been used to assess admission severity and/or effects of comorbidities of surgical patients. Garcia et al (2012) found that for each increase in ASA scores, average LOS (and therefore costs) increased [29] and Kay et al (2014) found the classification to be a reliable indicator for variance in LOS and total inpatient cost for hospitalised patients [30]. However, Shah et al (2004) previously found that ASA score was significant but only if a higher than score four (constant threat to life), so has limited usability for the bulk of the hospital population [13]. Notably, the ASA is only collected in surgical populations, which is roughly only 30% of the hospital population of persons aged over 50 years [31]. Admission severity is evidently a more difficult measure to establish, but a key contributor to length of stay and cost.

Episodes with hospital-acquired complications cost more than other episodes, after controlling for confounding factors [12, 32, 33]. Complications (including respiratory infection, urinary tract infection and pressure injuries) increase LOS by 20% [14], with pressure injuries responsible for over 30% of the increase in acute and total LOS. Similar complications average almost four times the LOS of separations than those without complications [15]. These common but relatively inexpensive (per case) complications, such as urinary tract infections and pneumonias, tend to cost the most to health systems because they are so common [34]. The combined cost of just four common hospital-acquired complications (urinary tract infections, pressure injuries, pneumonia and delirium) was found to be associated with 25% of the above-average length of stay in one Australian state among older adults, with eightfold increase in LOS, and double the estimated mean episode cost [22]. These four hospital-acquired complications are noted to be sensitive to nursing characteristics such as nurse staffing levels, education levels, skill mix and workload [35, 36]. Despite these costs, these four common complications are often overlooked when counting the costs of adverse events [37]; partly due to difficulty ascertaining what is hospital-acquired [37], and partly due to classification as geriatric syndromes rather than preventable complications [5].

### Summary of background

In Australia, public hospitals consume the largest component of health spending [38], older adults utilise the bulk of hospital bed days and associated funding [38] and patient safety efforts often focus on dramatic but rare or infrequent hospital-acquired complications [37]. Health administrators make decisions about priorities for expenditure and use information about what are fixed and what are potentially avoidable costs. Previous studies, including Jackson’s previous Australian costing study [37], are noted to overlook controls for compounding factors [39, 40]. Consequently more information is required on the relative costs associated with health characteristics common in older patients, as well as the influence of age itself. This study sought to understand the relative contributions of age, comorbid dementia, other comorbidities, admission severity and complications to patient length of stay and hospital cost in order to identify opportunities for cost containment and service improvement.

## Method

### Data source and coding

The present study was nested in the Australian Hospital Dementia Services Project [41–43], which uses hospital discharge data from the July 2006 to June 2007 Australian financial year for all public hospital overnight discharges for episodes of care for adults aged 50 and over (50+) in the Australian state of New South Wales (NSW). People aged 50+ were the chosen sample in order to be inclusive of young onset dementia and the Indigenous older population. Ages 50+ is considered ‘older’ primarily due to the gap in life expectancy compared to non-indigenous Australians. There were over 1.6 million discharges in the examined time period; excluding day-stays and patients under age 50 resulted in 426,276 discharges; including only those with above-average length of stay resulted in a sample of 157,178 discharges. NSW is Australia's most populous state with a diverse population from metropolitan to remote areas and a range of hospital-based and community-based dementia services.

Ethics approval to conduct the study was obtained from the NSW Population and Health Services Research Ethics Committee. Ethical approval above was based on the National Health and Medical Research Council (NHMRC) guidelines on ethical conduct in regard to waiver of consent; where it is impracticable to obtain an individual’s explicit consent to the use of their information. This study utilised administrative data for all-of-state hospital discharges, consequently consent to participate and consent to publish was waived, and all data deidentified in accordance with NHMRC and Privacy Act 1988 guidelines.

Using a person identifier, patients were coded as having dementia if dementia was ever documented as a principal or additional diagnosis (DRG ICD-10 codes include F00, F01, F02, G30, G31) in any hospital stay over a two-year period, 2005–2007, offering a high capture rate and minimising misclassification bias [42]. Using internationally valid patient-level and risk-adjusted coding rules for adverse outcomes [31, 35, 44], nurse sensitive hospital-acquired complications were identified. These nurse sensitive outcomes were identified through an expert review and then tested with over 6 million US hospital discharges [35], and confirmed in a later meta-analysis [9] to be patient outcomes that are directly or indirectly associated with nursing care quality and quantity. The coding rules are conservative, excluding patients at risk of developing complications due to their underlying aetiology, so that complications identified using these coding rules are likely to result from hospitalisation. For example, patients who have paralysis as a primary or secondary diagnosis are excluded from the complication ‘pressure injury’ because they are less mobile than other patients. Another example is that patients with a primary diagnosis related to the urinary tract are excluded from being coded as experiencing the complication ‘urinary tract infection’ because their aetiology predisposes them to the condition, making it more likely to be a disease rather than hospital-acquired complication.

For all complications, episodes with length of stay (LOS) beyond 90 days were excluded to maintain consistency of the approach to these complications with other approaches to administrative discharge data [44, 45]; this approach excluded 1,268 episodes, or 0.3% of the study population. Patients may have contributed multiple episodes to the same stay to the data set, but would only be collected if the complication was still coded by the clinical records department as contributing to the admission.

### Calculating the cost of length of stay

Publically available hospital data were used to calculate average LOS for each Diagnosis Related Group (DRG) for NSW for the financial year2006–07 [46]. Each DRG represents a class of patients with similar clinical conditions requiring similar hospital services. The average LOS for each DRG usually includes day-stay patients but, for this analysis, these short stays were excluded so that LOS could be compared with the study’s overnight population. Publically available hospital data were also sourced for the total average cost of patient discharges by DRG by state for the financial year 2006–07. NSW public hospitals provide estimates of costs by DRG broken down into treatment expense subcategories by age-group [47].

An established method using the cost subcategories was utilised to calculate which costs are dependent on LOS [48]. These costs can be ‘variable’ (ward nursing, ward medical, non-clinical salaries, pathology, imaging, allied health, pharmacy, supplies, on-costs (indirect salary costs such as superannuation and leave loading), hotel, depreciation) or one-off ‘fixed’ (critical care, operating rooms, emergency departments, special procedure suites, prosthesis). Variable and fixed costs were calculated separately for each DRG and each LOS, so that costs for each of the hospital episodes in the study were estimated (more detail of this process is available elsewhere [22]).

Patients with above-average LOS are of interest because the excess component of LOS is modifiable and may be responsive to interventions. Consequently, this was chosen as the dependent variable for this study (similar approaches used to examine prolonged length of stay can be seen in other studies: [27, 49–51]). Patients who stayed longer than the all-ages overnight state average for their DRG were considered to have ‘above-average’ LOS. The state-average LOS was subtracted from these patients’ LOS to calculate the number of additional days that each ‘above-average’ patient stayed. To calculate the ‘extra costs’ for these patients, their additional days were multiplied by the daily *variable* cost for their DRG (i.e., excluding the one-off fixed costs that do not change with LOS).

### Analytic approach

The Charlson Index was derived to provide a summary measure of patient comorbidity based on the presence of diabetes, hemiplegia or paraplegia, any cancer, HIV/AIDS and major cardiovascular, renal, rheumatic, peptic ulcer and liver diseases [52]; dementia would usually also be included in the Charlson Index but was excluded due to being a discrete variable for investigation in this study. To represent admission severity, ‘mean cost weights’ were utilised from state data. Cost weight is a measure of the estimated average cost of an DRG benchmarked against the average cost of all separations [46]; this measure reflects illness severity and resource use. In this study, the state cost weights were applied to the study patient episodes according to their DRG to account for patient admission severity. All analyses were conducted using SAS EG V.9.2 with records with missing data excluded from analysis as required. Level of significance was set at 5%. Population characteristics for patients withabove-average length of stay (N = 157,178) were 11.9% with dementia; 14.2% with a key complication; 53.0% female; and 23.7% had surgery. Age range was 50–107 years. Overall complication rates were: 8.2% urinary tract infection; 2.6% pressure injury; 3.7% pneumonia; and 1.9% delirium, comprising a total of 14.6% experiencing any one of these four complications. Multiple complications were infrequent; with 0.87% of the population experiencing any two of the complications, 0.07% any three; and 0.01% experienced all four complications. Other characteristics are presented in Table 1.

**Table 1:** Main characteristics of continuous variables.

Continuous variables	Mean	Minimum	Maximum
Admission severity (cost weight)	1.9	0.1	46
Comorbidity (Charlson Index)	1.1	0	13
Age (years)	73	50	107
Extra cost (A$)	5,875	51	114,486
Complications (number of)	0.1	0	4

A hierarchical step-wise selection procedure was used in a linear regression model to predict cost of excess length of stay, controlling for different kinds of patient case complexity. Age (50–107) and sex (M/F) were added in the first step followed in the second step by surgery (Y/N), dementia (Y/N), admission severity (cost weight 0–46), comorbidities (Charlson Index 0–13) and number of complications (1, 2, 3 or 4). The data did not fully meet the assumptions of the analyses. There was evidence of heteroscedasticity in the residuals in the final model. The variables admission severity, complications and comorbidities were positively skewed; and the dependent variable, excess LOS, was also positively skewed, such that skew was 0.4, with a minimum score of $51, maximum of $114,486 and mean of $5,815. There was also some limited multicollinearity among the independent variables (e.g., surgical status was moderately correlated with cost weight; *r* = 0.40). In response, the dependent variable, excess cost, was logtransformed to a natural logarithmic scale. Though this was useful, standard errors cannot be accurately reversed from a log transformation and cannot therefore be reported [53]. Instead, *standardised beta coefficients* are presented to show the relative contribution of each predictor variable to explaining variance in LOS in the final model. To address the skew in age in years, the use of age brackets was tested (50–65, 65–74, 75–84, 85+). As this did not improve the model fit, age in years was used.

## Results

The first step model with only age and sex explained a trivial proportion of the variance in extra cost (R^2^ = 0.017). Adding surgery, dementia, admission severity, comorbidities and number of complications in the second step of the model explained 13% of variance in extra cost. The model revealed that each hospital-acquired complication predicted an extra cost of $812 (US$1,159 converted using ‘purchasing power parity’ for 2006 and 2007 ratios from www.imf.org), when extra cost was also explained by dementia, other comorbidities, admission severity, age, sex, and surgical status (Table 2). Dementia contributed significantly to explaining variance in extra costs, such that dementia cost of $873 (US$1,247) more than non-dementia when all other variables were held fixed. Other comorbidities contributed significantly to explaining variance in extra costs such that having a comorbidity index of one cost $259 (US$370) more than having no comorbidity. Similarly, each increase in admission severity score explained the variance in extra costs by A$295 ($US421). In the final model, sex contributed significantly to explaining variance in extra costs such that men cost $87 more than women. Admission severity and comorbidity had higher relative importance in predicting extra LOS, and they were better predictors of variance than the other measures of complexity (see the standardised coefficient in Table 2). So for adults over 50 who have length of stay longer than average for their diagnostic condition, comorbid dementia predicts extra costs (all estimates significant at *p<*0.0001). Hospital-acquired complications and dementia cost more than other kinds of inpatient complexity, but patient admission severity was a better predictor of excess cost.

**Table 2:** Statistics derived from linear regression analysis including estimate of percentage of costs.

Explanatory Variables	Coefficient (unstandardised) (B) in $A (Reverse logtransformed)	Beta (standardised coefficient)	Percentage of average Extra LOS costs^
Dementia	873*	0.09	15%
Complications	812*	0.10	14%
Admission severity (cost weight)	295*	0.22	5%
Comorbidity (Charlson Index)	259*	0.15	4%
Age	20*	0.09	0%
Surgical status (surgery)	49*	0.01	1%
Sex (male)	87*	0.02	1%

R^2^ = 0.13; Adj. R^2^ = 0.13; *F*-Value (3445.8)

N = 157,178

* = *p*<0.0001

^$A 5875 is the average cost of above-average LOS for over 50s [22]

**Fig 1:**
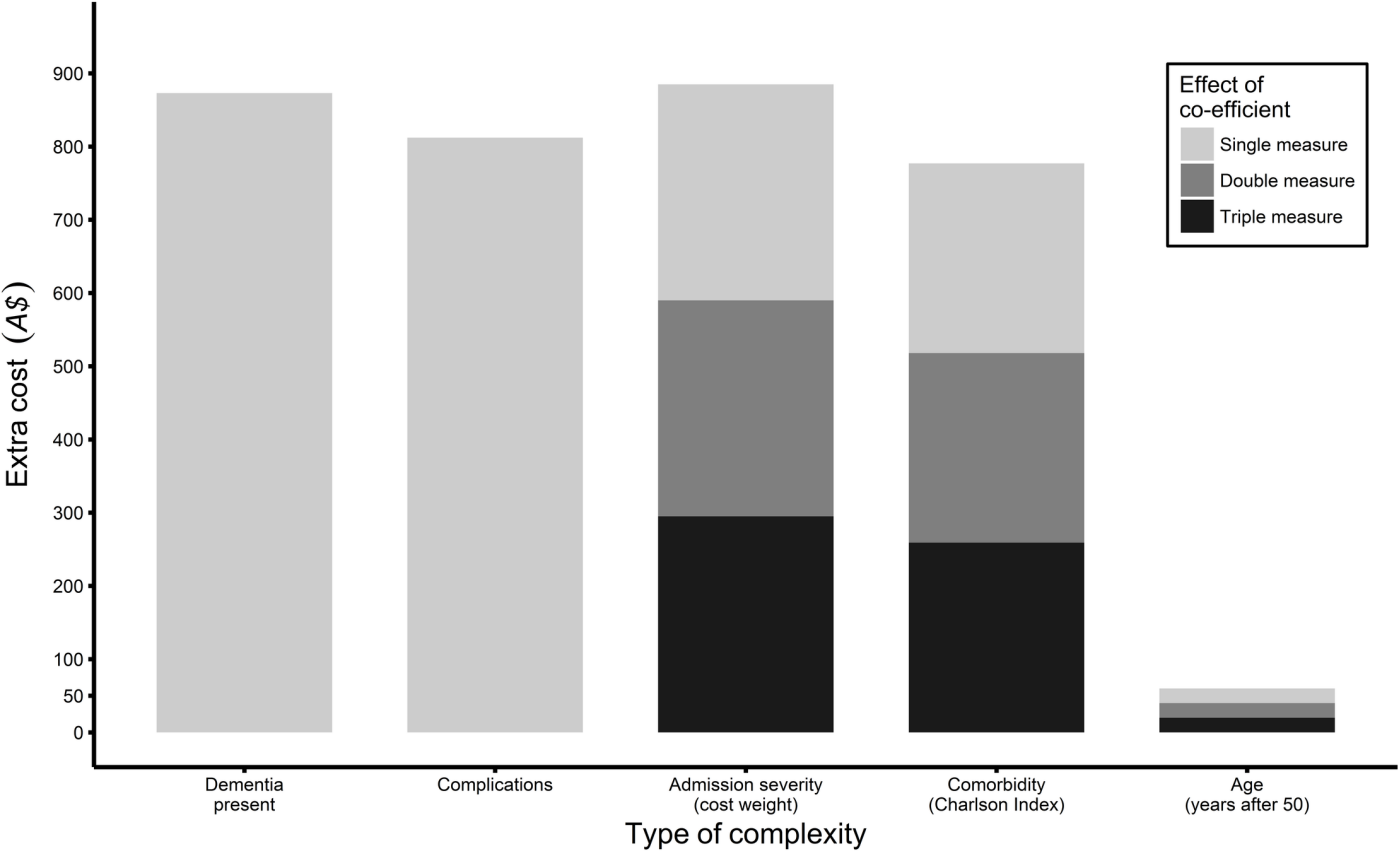
Effect of coefficient.

Dementia and any one hospital-acquired complication were equivalent to 15% and 14% respectively of the mean extra LOS costs for the study population. Whereas, admission severity and other comorbidities were equivalent to 5% and 4% of the average extra LOS costs. Consequently, as shown in Fig 1, patients would need to have three times the admission severity score or comorbidity index to generate equivalent extra costs as having dementia or one complication. Year of age, sex or surgery had minimal impact on extra cost.

## Discussion

### Summary of research findings

This study set out to examine the predictors for extra cost for adults aged over 50 years in hospital. The study found that when all other factors (complications, other comorbidities, admission severity, age, sex and surgical status) are held constant, dementia predicts an extra cost of $873. Complications were also more costly than other kinds of complexity when they were held constant, predicting extra costs of $812. However, the standardised coefficient highlighted that admission severity, followed by comorbidities other than dementia, were better predictors of variance than the other variables considered.

### Comparison with previous research

Dollar cost comparisons with other international studies is hampered by different accounting methods, dollar equality and health services approaches. However, this study reinforces other findings that common, potentially preventable hospital-acquired complications, that are known to be sensitive to nursing care, are recognised as a key contributor to extra LOS costs [12, 34, 54]. This study also confirms that inclusion of a range of key variables is required when examining cost and LOS, and that these include comorbidities, admission severity and age [11, 28, 55]. What is a key finding is the complex interplay between complications and dementia, and their high impact on extra LOS and cost. Previous work has highlighted that complications affect costs differently for patients with and without dementia: adults with dementia are more likely to suffer a complication, but once they do, they are more likely to have a cheaper cost that non-dementia patients [23, 31]. Examination of the interplay specifically between complications and dementia was outside the scope of the present study but, given the potential importance for costs and clinical approaches, future studies should utilise a mechanistic experimental approach to explicitly examine it [5, 56–58]. The finding that admission severity explains variance in use of hospital care (and therefore cost) is reinforced elsewhere [59].

### Theoretical implications of findings

The health field is undergoing considerable developments regarding the positioning of preventability, risk and safety in light of long-term efficiency and sustainability [5, 24, 60]. Hospitalised elders are particularly susceptible to infectious complications as they often have multiple chronic diseases, require longer periods of hospitalisation and have more frequent use of invasive devices [61]. Moreover, many of the standard interventions being delivered in hospital are seen as unnecessary, and perhaps even contributing to complications in aged and dementia populations [62]. Pressure injuries and delirium are commonly researched as ‘geriatric syndromes’[63], but the growing evidence suggests that, when occurring in hospital, these are more usefully considered as adverse events related to care rather than a common collection of symptoms [5, 37, 64]. The bourgeoning research into dementia in acute care, where the rate of publications was ten times higher in 2012 than in previous years [65], is another indicator that researchers are seeking better information on how to evaluate quality, estimate cost and provide care and treatment to the complex, older population. The findings of this study offer cost implications to contribute to the construction of the problem; that length of stay for older patients cannot be understood, and therefore better managed, without understanding the key components and interplay of complexity.

Recognition of complexity will need to make it to funding systems, and are unlikely to be represented by current frameworks that are disease, rather than symptom, focused. Severity of illness is a key moderator of patient demand for care and therefore resource use [66]. Pugely et al. (2014) comment that reimbursement systems using ‘complications and comorbidity’ are unlikely to renumerate for many of the complexities that contribute to increased LOS and other costs, and that increasing numbers of patient comorbidities were associated with compounding increases in resource use [9]. [59] found that inclusion of medical or nursing diagnosis increased the explanation of resource use in hospital, but also that the organisational and staffing characteristics also explained the models–indicating that any models investigating costs will be incomplete without these key variables. These models of care and organisation of health care professionals are key in the recognition, and appropriate reimbursement strategies, of efficiencies of scale. Wilson et al (2014) found that, in caring for older patients, hospitals with higher average admission severity were associated with higher quality, and also associated with higher costs, indicating that hospitals may benefit from a critical mass of complex patients in order to develop economies of scale in the delivery of quality care [25]. Further research using bottom-up costing that investigates severity of illness and intensity of care is required to better understand this issue. Frailty, palliative care, cognitive impairment and physical impairment all have complexity costs of labour which may not be well recognised in current costing models which perceive care labour as an overhead cost. These kinds of broader support and care complexities increase with the success of longevity and accumulation of comorbidities; these complexities are also remarkably heterogeneous in the ageing population (the ‘sick old’ versus the ‘fit old’[67]). Nursing intensity costs are invisible in current costing models which utilise medical complexity related to DRGs. Complex calculations based on DRGs are utilised to try to derive functional care requirements and nursing workload complexity needs of inpatients.

### Limitations

Administrative data is limited in accuracy, both to identify adults with dementia, and to identify complications. The comprehensive linked approach used here means that dementia is better captured than in many similar studies. Future studies could benefit from breaking down analysis into dementia types to better understand drivers of admission and costs of care during admission. The patient-level risk-adjustment model to capture complications also offers a degree of accuracy not seen in similar administrative data studies. Future use of the ‘condition onset flag’, established in NSW since this study, will improve accuracy. The location of NSW offers a fair representation of Australian adults with dementia, but may not represent other countries. In reviewing length of stay (LOS), it is important to remember that it is an incomplete outcome measure, and indeed, an inappropriate measure of quality. Firstly, because LOS should be patient focused and is not necessarily aligned with quality outcomes. Secondly, LOS has most costs associated with the front end of LOS [18, 68]; though the distinction between variable and fixed costs should alleviate this. However, for examining outcomes for a large population, LOS remains a simple and relatively useful measure.

Sampling only above-average LOS compared to the patient’s DRG is a novel approach to examine potentially modifiable patient experiences. This approach may be useful in future examination of potentially unnecessary or unwanted delays or complications, including social and logistic reasons such as awaiting placement or support interventions for discharge. However care needs to be taken in interpretation, given that half the sample (those with below average LOS) is excluded. Additionally, this study uses cost weight as a proxy for patient admission severity, but it should be noted that cost weight only uses medical complexity related to DRGs–it does not include functional ability nor nursing workload complexity. The increasing focus of administrative data to capture complexity in upcoming hospital costing system variations is welcomed, however the inclusion of functional impairment would further improve models to predict length of stay and cost but may not be feasible [69]. Inclusion of functional (and indeed, cognitive) impairment in hospital data collection would also offer a proxy for nursing workload, and is particularly significant in complexity of older patients [70].

The model fitted in this study resulted in a small R^2^, though this is not uncommon in health services research [27] where contributing variables are numerous. Those studies with over 100 variables may offer greater prediction but lower usability [55]. The effects seen in this study provide important insight into clinical practice. Better understanding of the complexity of the aged population, as well as the complexity of hospital care delivery systems, particularly given the increasing life expectancy and increasing comorbidity of the population that hospitals service, is crucial to future health services research and economic decision making.

### Future research

Recognition of the complexity of the hospitalised older population is necessary and requires further research. Consideration of cost/benefit may include release of the A$225 million identified as potentially avoidable extra costs [22] for the four complications in order to pay for initiatives to prevent those complications. Nurse staffing mix, age-friendly acute care models and healthy work environments show promise in risk reduction for these potentially preventable complications [35, 71–74], but warrant further application and evaluation. Other research models which could be evaluated in terms of reduction in cost of complications and dementia for complex, older patients include ‘cascade iatrogenesis’ [75], associations with implicit care rationing by nurses [76, 77] and relationships between functional decline and models of care for the aged in hospital [4, 78, 79]. Increasing the cost evaluation within these approaches, and including variables that control for nursing workforce characteristics, is supported by the gerontology literature [59].

### Practical implications of the research

Of the patient complexities found here to be associated with extra costs, how dementia patients are managed and how complications are mitigated in hospital are modifiable. The high-frequency complications, seen more often in adults with dementia, offer the greatest opportunity to decrease hospital costs by decreasing incidence, and improving early detection and management. Older, more complex patients are becoming the largest consumers of hospital beds, and acute hospitals are the largest consumers of health budgets. As with any other health condition, if these patients are at higher risk of complications, then it is the health service’s responsibility to mitigate those risks. Otherwise they are high-risk patients with no prophylaxis.

## Conclusion

These findings reveal that while admission severity of patient episode is the better predictor of cost of extra length of stay, any complication or dementia are contributing similar amounts to an admission cost. Patients would need to have three other kinds of comorbidities to have an excess length of stay cost equivalent to a dementia diagnosis. Complications and dementia both have modifiable care components for patients while in hospital, particularly related to the organisation of nursing and models of care. Identifying patients who are high-risk, and then putting strategies in place to modify their risk profile, is a normal part of hospitalisation and should be extended to the elderly population to cover all aspects of complexity.

## Abbreviations

AIHW: Australian Institute of Health and Welfare
ASA: American Society of Anesthesiologists’
DRG: Diagnosis Related Group
HIV/AIDS: Human Immunodeficiency Virus/Acquired Immune Deficiency Syndrome
ICD: Internationally Classification of Diseases
LOS: Length of stay
NSW: New South Wales
$A: Australian dollars
$US: United States of American dollars
